# Development and validation of predictive risk models for sight threatening diabetic retinopathy in patients with type 2 diabetes to be applied as triage tools in resource limited settings

**DOI:** 10.1016/j.eclinm.2022.101578

**Published:** 2022-07-22

**Authors:** Manjula D. Nugawela, Sarega Gurudas, A. Toby Prevost, Rohini Mathur, John Robson, Thirunavukkarasu Sathish, J.M. Rafferty, Ramachandran Rajalakshmi, Ranjit Mohan Anjana, Saravanan Jebarani, Viswanathan Mohan, David R. Owens, Sobha Sivaprasad

**Affiliations:** a UCL Institute of Ophthalmology, 11-43 Bath Street, London EC1V 9EL, United Kingdom; b Moorfields Eye Hospital NHS Foundation Trust, London, United Kingdom; c King's College London, Nightingale-Saunders Clinical Trials and Epidemiology Unit, London SE5 9PJ, United Kingdom; d London School of Hygiene & Tropical Medicine, Keppel Street, London WC1E 7HT, United Kingdom; e Queen Mary University of London, Institute of Population Health Sciences, London, E1 4NS Wales, United Kingdom; f Swansea University Medical School, Swansea University, Singleton Park, Swansea, Wales SA2 8PP, United Kingdom; g Madras Diabetes Research Foundation and Dr. Mohan's Diabetes Specialities Centre, Chennai 600086, India; h Population Health Research Institute, McMaster University, Hamilton, ON, Canada; i Department of Primary Care and Public Health, Imperial College London, London, UK

**Keywords:** Diabetic, Retinopathy, Predictive models, Performance, Diabetes, South Asians, India, BMI, Body mass index, CCG, Clinical Commissioning Group, CI, Confidence Interval, CPRD, Clinical Practice Research Datalink, CVD, Cardiovascular disease, DR, Diabetic Retinopathy, GP, General Practice, HR, Hazard ratio, OR, Odds ratio, NHS, National Health Service, STDR, Sight threatening diabetic retinopathy, T2DM, Type II diabetes mellitus, UK, United Kingdom

## Abstract

**Background:**

Delayed diagnosis and treatment of sight threatening diabetic retinopathy (STDR) is a common cause of visual impairment in people with Type 2 diabetes. Therefore, systematic regular retinal screening is recommended, but global coverage of such services is challenging. We aimed to develop and validate predictive models for STDR to identify ‘at-risk’ population for retinal screening.

**Methods:**

Models were developed using datasets obtained from general practices in inner London, United Kingdom (UK) on adults with type 2 Diabetes during the period 2007–2017. Three models were developed using Cox regression and model performance was assessed using C statistic, calibration slope and observed to expected ratio measures. Models were externally validated in cohorts from Wales, UK and India.

**Findings:**

A total of 40,334 people were included in the model development phase of which 1427 (3·54%) people developed STDR. Age, gender, diabetes duration, antidiabetic medication history, glycated haemoglobin (HbA1c), and history of retinopathy were included as predictors in the Model 1, Model 2 excluded retinopathy status, and Model 3 further excluded HbA1c. All three models attained strong discrimination performance in the model development dataset with C statistics ranging from 0·778 to 0·832, and in the external validation datasets (C statistic 0·685 – 0·823) with calibration slopes closer to 1 following re-calibration of the baseline survival.

**Interpretation:**

We have developed new risk prediction equations to identify those at risk of STDR in people with type 2 diabetes in any resource-setting so that they can be screened and treated early. Future testing, and piloting is required before implementation.

**Funding:**

This study was funded by the GCRF UKRI (MR/P207881/1) and supported by the NIHR Biomedical Research Centre at Moorfields Eye Hospital NHS Foundation Trust and UCL Institute of Ophthalmology.

**Research in context**

**Evidence before this study**

We searched PubMed and Google Scholar for studies published from January 1980 to June 2021 using search terms (“diabetic” or “diabetes” or “T2DM”) AND (“retinopathy” or “maculopathy”) AND (“prediction” or “progression” or “risk model”) and found several prognostic models for sight threatening diabetic retinopathy (STDR). However, most of them require previous record of retinopathy status, glycated haemoglobin (HbA1c) estimation or other clinical or laboratory parameters and therefore it is difficult to use these models in in low-resource settings.

**Added value of this study**

We have developed and externally validated three risk prediction equations to estimate the absolute risk of STDR over a period of 3 years. These models are ideal for use in low resource settings as they are limited to demographic and clinical parameters that patients can inform fieldworkers in community screening. All three models are well calibrated and have good performance with C statistics close to 0.7 and above in both the development and external validation datasets. Our risk prediction equation and the risk charts may be used to stratify people with diabetes at risk of STDR from the community for urgent retinal screening especially in countries with no established systematic diabetic retinopathy screening programmes.

**Implications of all the available evidence**

Retinal screening is recommended for all people with diabetes at a regular basis but people living in many low- and middle-income countries do not have access to systematic screening. These models have the potential to identify those who need prioritisation for retinal screening and treatment whilst health systems are being improved to accommodate systematic retinal screening for all people with diabetes. Further validation and piloting of these models are required before implementation at community level or for self-assessment.

Alt-text: Unlabelled box

## Introduction

Diabetes and its complications are a significant global health burden and currently, there are around 463 million people with diabetes worldwide.^1^^,^^2^ Diabetic retinopathy, the most common microvascular complication of diabetes, is a common cause of visual impairment in the working age group people.^2^^,^^3^ The retinopathy can progress to sight threatening diabetic retinopathy (STDR) without any symptoms and STDR has to be identified early by retinal examination or photography.^4^ It is estimated that approximately 28 million people with diabetes have STDR globally.^5^ Therefore, systematic regular retinal screening is recommended for all people with diabetes. However, retinal screening is resource intense, and most countries do not have the expertise or facilities to develop and sustain a systematic retinal screening programme for their increasing population with diabetes. Due to other pressing health priorities, establishing diabetic retinopathy screening is not a priority in the majority of less-developed countries. Therefore, the numbers of people with visual impairment due to STDR is likely to increase with the rising prevalence of diabetes. There is an unmet need to identify those at risk of STDR using easily available predictors so that they can be prioritised for retinal examination or screening. In addition, a targeted optimisation of risk factors for this group of individuals may also reduce the risk of disease progression.

There are several prognostic models that have been developed for STDR to personalise retinal screening strategy.^6^^,^^7^ However, these cannot be applied in low-resource settings as most of them require previous record of retinopathy status, glycated haemoglobin (HbA1c) estimation or other laboratory or clinical parameters (Supplement Table 1).^8^, ^9^, ^10^, ^11^, ^12^, ^13^, ^14^, ^15^, ^16^, ^17^, ^18^, ^19^, ^20^, ^21^, ^22^, ^23^, ^24^, ^25^ Therefore, although the presence of diabetic retinopathy and HbA1c are strong predictors of STDR, an ideal risk model for STDR in low resource settings should be limited to demographic and clinical parameters that patients can inform fieldworkers in community screening. Community-based health-screening is widely practised in low and middle-income countries as primary care is still in its infancy.

Although systematic diabetic retinopathy screening with retinal camera is the gold standard, most people with diabetes do not have access to this service globally. The aim of this study was to develop predictive models for STDR that could be applied based on available resources so that those at risk of STDR could be prioritised for retinal screening from the growing population with diabetes.

## Methods

Local research ethics approval was obtained from Moorfields Research Management Committee. Further ethics approval from Health Research Authority was not required as the study included only fully anonymised data. Approval was also obtained from the Caldecott guardian of these anonymised datasets in Queen Mary University London (QMUL) and Secure Anonymised Information Linkage (SAIL) in Wales and local research ethics approval in Madras Diabetes Research Foundation (MDRF), Chennai, India. This study was conducted in accordance with the Declaration of Helsinki. Patient-level consent was not required as the study only used fully anonymised routinely collected data (SIVS1057, Moorfields Eye Hospital dated 14/04/2020).

### Study design, setting and data source

#### Model development dataset

We developed the predictive models using existing dataset obtained from general practices (GP) in three Clinical Commissioning Groups (CCGs) in East London, which included Newham, Tower Hamlets, and City and Hackney. The dataset covered more than 98% of the GP-registered multi-ethnic population in these CCGs. The data included demographic information, diagnoses, prescriptions, referrals, laboratory test results and clinical values. Diagnoses, symptoms and clinical values were recorded using read code classifications.

We included all adults with a diagnostic read code for type 2 diabetes (T2DM) during the period 01/01/2007-31/12/2017 and aged 18 or over at study entry. As the retinal screening events and DR screening events were not recorded simultaneously, we allowed for a 6-month delay for DR events to be recorded from the date of retinal examination. Study baseline was defined as the date of the first DR screening examination (or recorded non-STDR event date where retinal screening examination was not recorded in the previous 6 months) within the study start and end dates. Start date was defined as the latest of 01/01/2007, the patients registration date, or the date the patient turned 18. In our cohort, participants need a baseline screening episode or DR event followed by a last screening episode or DR event recorded during the study period. However, participants who developed STDR outside of the study period, require their last screening episode or last non-STDR event date not necessarily to have occurred during the study period but recorded at least 6 months prior to their STDR onset date. Follow-up time end date is defined as the earliest date of first diagnosis of STDR, date of death, 31/12/2017, de-registration from the GP, or the date of their last DR screening appointment or last DR event. People who were lost to follow up were censored at the date they left the study. Follow-up time was censored at 3-years.

### Model validation datasets

Fully anonymised data from the SAIL databank^26^ was used for external validation, consisting of 170,588 T2DM participants registered with over 170 primary care-GP practices, successfully screened for DR from Wales between 2008 and 2017, of predominately White ethnicity. The SAIL databank record absence of DR (R0) and absence of Maculopathy (M0) unlike the development cohort and screening results are recorded alongside the screening appointment. Entry of individuals into this cohort was at any time during an 11-year period from 01/01/2008-31/12/2017. The cohort entry date was defined as the latest of date of 18^th^ birthday, date of registration with the GP or 01/01/2008. Follow-up time ended at the earliest of date of 31/12/2017, de-registration from the GP, death, onset of STDR or the date of their last successful DR screening appointment. For participants who developed STDR outside of the study period, a recorded screening episode prior to the event and after study end was required to ensure they were disease free by study end. Index date or study baseline was defined as the date of first DR screening appointment between cohort entry and end of follow-up. The second external validation cohort included individual participant hospital data from Dr Mohan's Diabetes Specialities Centre (DMDSC) and the Madras Diabetes Research Foundation (MDRF), a tertiary care diabetes hospital, Chennai, India that screens all people with diabetes registered in the hospital on a regular basis. The DMDSC MDRF Diabetes Electronic Medical Records (DEMR) system identified 19,909 individuals with T2DM who had undergone routine screening for assessment of DR and eGFR during 2011 and followed-up regularly every 3–6 months between 2011 and 2018.

### Outcome

The main outcome was STDR and this was classified according to the American Academy of Ophthalmology International Classification as the first record of severe non-proliferative diabetic retinopathy, proliferative diabetic retinopathy or diabetic macular oedema. In the UK datasets, the respective grades of R2, R3 and M1 based on the English Diabetic Eye Screening Programme classification were used. The grades of retinopathy were determined by trained graders or ophthalmologists from retinal images captured through dilated pupils using fixed retinal cameras.

### Predictor variables

Based on existing literature (Supplement Table 2) we have identified the predictors that are found to be associated with the outcome and then these variables were checked in the model development dataset. After considering their availability in the model development dataset we considered the following risk factors in this study: age, HbA1c, systolic blood pressure (SBP), duration of diabetes, body mass index (BMI), total cholesterol, antidiabetic medication, estimated glomerular filtration rate (eGFR), history of cardiovascular disease (ischaemic heart disease, heart failure, stroke, peripheral vascular disease, cardiovascular death, acute myocardial infarction, bypass graft/angioplasty, angina pectoris, cardiac arrhythmia, major ECG abnormality, silent myocardial infarction, congestive heart failure, transient ischemic attack, arterial event requiring surgery). These covariates were measured at baseline for each individual and the closest record to the index date (± 6 months) was selected for clinical variables.

The covariates considered in the SAIL dataset were also recorded in the same way as the model development dataset, taking the closest record to the index date (± 6 months) and coding standards were consistent with Quality outcomes Framework (QOF).^27^ However, eGFR in the SAIL dataset was calculated from data on serum creatinine, ethnicity and age using the 4-variable Modification of Diet in Renal Disease Study equation unlike in the model development dataset where eGFR was directly provided by the laboratories.^28^ The MDRF dataset had fewer newly diagnosed participants as patients were seen in secondary care (hospital data), which may or may not have been the initial point of contact unlike the UK primary care datasets. Eligibility criteria for MDRF was different to that of the UK cohorts, in that the dataset was previously curated to include participants who had concurrently undergone routine screening for assessment for eGFR and DR.^29^

#### Sample size and missing data

The Events per Variable (EPV) was between 18 and 95 in the datasets, assuming a maximum number of 35 parameters used in this study, indicating adequate sample size for model development and validation.^30^ All covariates were inspected for missing values by coding the missing data as a separate category and then they were considered in the univariable and multivariable modelling. None of the variables retained in the final models had missing data and therefore, no further actions were required.

### Model development and validation

We developed the predictive model using the Cox proportional hazard model given below.

$$h\left(t\right)=h_{0}\left(t\right)\text{exp}\left(\beta_{1}x_{1}+\beta_{2}x_{2}+\ldots +\beta_{n}x_{n}\right)$$

where:

t – time from start date
h(t) – hazard function
h**_0_** (t) _–_ Baseline hazard function
β_1 -_ Coefficient for predictor x_1_
β_2 –_ Coefficient for predictor x_2_
β_n –_ Coefficient for predictor x_n_

Predictive factors were selected using backward elimination procedure while considering statistical significance at each step, variables that were statistically insignificant (*p>*0.05) were removed until all of the variables became statistically significant in the model (*p<*0.05). Final parsimonious model was then further assessed for its performance in the development data set and then each variable was assessed for its contribution towards model performance, those variables with least contribution (<0.1 change in C-statistic) were removed from the model and this was identified as Model 1. A further reduced model was developed by removing predictors that would require laboratory testing and retinal screening expertise and therefore difficult to apply in resource restricted settings (Model 2). A minimal model with non-invasive predictors was then obtained by removing any clinical variables/laboratory tests from reduced model above and this was named as Model 3. These three models were used to assess the effects of prognostic factors, and hazard ratios (HR) with 95% confidence intervals (CI) are presented for each of the variable.

Internal validity of the model was assessed according to the measures of model performance in the development datasets.^30^ External validity of the model was assessed in SAIL and MDRF datasets described above. For both internal and external validation, model performance was assessed using calibration and discrimination measures where calibration, is the agreement between observed and predicted times to the outcome.^31^ The calibration slope was quantified using the beta-coefficient of the linear predictor in each dataset. This gives an impression of whether risks were over or under predicted across all time points. To visualise calibration at a single time-point at 3 years across various risk thresholds, participants were categorised into deciles of 3-year STDR risk, with observed (3-year Kaplan-Meier event rate) and mean predicted risks plotted for each group. The ratio of observed to expected (O/E) risks were also reported which summarises the agreement between Kaplan-Meier event rate and mean predicted risk at 3 years.^31^ Model discrimination is the ability of the model to differentiate between patients who reached the endpoint and those who didn't. This measure is quantified using the C-statistic in other words Area Under the Curve. A C–statistic between 0·6 up to 0·7, and a C-statistic between 0·7 and 0·9 suggests good and strong discrimination of the predictive models respectively. Models were updated in each external validation cohort, if required, by re-calibrating the baseline survival function at 3-years. This is where the calibration slope is set to 1, by assigning the linear predictor as an offset term in the model for the external validation datasets. Updating of the baseline survival aims to correct the calibration slope to make the average predicted risk equal to the observed overall event rate. A risk chart was developed for the minimal model (Model 3) using colours representing high, medium and low risk for having STDR within the next 3 years. This chart was produced using all three datasets, model development and model validation datasets in the UK and India. Data management and analysis was performed using Stata 17 (Stata Corp., College Station, Texas, USA).

### Sensitivity analysis

As the date of onset of STDR is difficult to pinpoint with routine data, final models were re-estimated accounting for the interval censored nature of retinal screening data using the interval-censored Cox model.^32^^,^^33^ These models were fit using the *IcenReg* package in R.^34^ In this approach the left interval was defined as the time from baseline to the last screening examination in which the participant was event free and the right interval the time from baseline to the date STDR was recorded. Participants who did not develop STDR were assumed to be right censored where previous definitions for end of follow-up apply. DR events recorded within 6 months of their retinal screening date were assumed to be the result from that screening visit for the model development dataset as screening and DR events were not recorded simultaneously. Moreover, incidence rates generated from the nonparametric Turnbull's estimator^35^ (allowing for events to occur in an interval) using the R package *Interval*^36^ and Kaplan-Meier estimate (assuming specific event times) were compared.

Role of the funding source: The funder had no role in the design and conduct of the study; collection, management, analysis or interpretation of the data; preparation, review or approval of the manuscript; and decision to submit the manuscript for publication.

### Ethics approval and consent to participate

Ethical approval was not necessary for the use of deidentified data derived from routinely recorded information in the electronic health record accessed by the Clinical Effectiveness Group Queen Mary University of London with the permission of the general practitioner data controllers.

## Results

The model development dataset included 71,908 people with T2DM diagnosis and screening code during the period from 2007-2017. From these patients after several exclusion criteria as explained in detail in Supplement Figure 1 there were 40,334 patients eligible to be included in the study.

### Overall study population

Table 1 shows baseline characteristics of study population by each dataset. Overall, 40,334 people in model development dataset, 102,672 people in UK validation dataset and 17,509 people in Indian validation dataset met our inclusion criteria. All of the variables that contributed toward the final models (age, gender, duration of diabetes, HbA1c, history of background or mild to moderate retinopathy) were complete in the model development dataset and ethnicity was recorded among more than 98% of the model development dataset.

**Table 1:** Baseline characteristics of the population in three datasets.

	Development Dataset	UK Validation Dataset	India Validation Dataset
	*N* = 40,334	*N* = 102,672	*N* = 17,509
**Duration of diabetes at baseline**^a^			
0-2 years	21,672 (53.7%)	57,352 (55·9%)	2395 (13.7%)
2-5 years	6503 (16.1%)	17614 (17·2%)	2764 (15.8%)
5-10 years	6542 (16.2%)	19018 (18·5%)	4836 (27.6%)
>10 years	5617 (13.9%)	8688 (8·5%)	7514 (42.9%)
**Duration of diabetes, years Median(IQR)**	1.5 (0.3-6.2)	1.4 (0.3-5.4)	8.2 (4.0-13.5)
**Age at baseline, years**			
<45	9211 (22.8%)	8143 (7.9%)	3771 (21.5%)
45-54	11229 (27.8%)	17,067 (16·6%)	5974 (34.1%)
55-64	9471 (23.5%)	28,311 (27·6%)	5520 (31.5%)
65-74	6892 (17.1%)	29,903 (29·1%)	1902 (10.9%)
75+	3531 (8.8%)	19,248 (18·7%)	342 (2.0%)
**Age, years mean (SD)**	55.7 (13.2)	63.5 (12.5)	53.4 (10.2)
**Gender**			
Male	21,676 (53.7%)	58,124 (56·6%)	11,440 (65.3%)
**Ethnicity**			All Indians
White	10,572 (26.2%	46,182 (45.0%)	
South Asian	18,913 (46.9%)	971 (0·9%)	
Black	8161 (20.2%)	261 (0·3%)	
Other	2291 (5.7%)	775 (0·8%)	
Not recorded	397 (1.0%)	54,483 (53·1%)	
**Body Mass Index (kg/m^2^)**			
<18·5	453 (1.1%)	221 (0·2%)	99 (0.6%)
18·5 – 25	6745 (16.7%)	9096 (8·9%)	5710 (32.6%)
25-30	14,581 (36.2%)	28,440 (27·7%)	8077 (46.1%)
≥30	16,251 (40.3%)	55,493(54·0%)	3623 (20.7%)
Not recorded	2304 (5.7%)	9422 (9·2%)	0 (0.0%)
**BMI, kg/m^2^ mean(SD)**	30.0 (6.1)	32.3 (6.6)	27.0 (4.1)
**HbA1c (mmol/mol)**			
<50	12,972 (32.2%)	41,591 (40·5%)	3776 (21.6%)
50 to 59	12,681 (31.4%)	32,952 (32·1%)	4169 (23.8%)
60 to 69	6082 (15.1%)	13,396 (13·0%)	3084 (17.6%)
70 to 79	3289 (8.2%)	6030 (5·9%)	2311 (13.2%)
80 and over	5310 (13.2%)	8703 (8.5%)	4169 (23.8%)
Not recorded	0 (0.0%)	0 (0.0%)	0 (0.0%)
**HbA1c, mmol/mol, mean(SD)**	60.3 (18.7)	56.5 (15.8)	67.0 (20.3)
**Systolic Blood Pressure (mmHg)**			
<120	9033 (22.4%)	11,832 (11·5%)	3609 (20.6%)
120-129	10,148 (25.2%)	20,835 (20·3%)	4731 (27.0%)
130-139	10,742 (26.6%)	29,938 (29·2%)	4245 (24.2%)
140-149	5816 (14.4%)	23,161 (22·6%)	2708 (15.5%)
150-159	2146 (5.3%)	7395 (7·2%)	1171 (6.7%)
160 and over	1893 (4.7%)	7038 (6.9%)	1039 (5.9%)
Not recorded	556 (1.4%)	2473 (2.4%)	6 (0.03%)
**Systolic blood pressure, mmHg, mean(SD)**	130.0 (16.1)	134.5 (15.6)	129.2 (16.4)
**Total Cholesterol (mmol/L)**			
<5·2	29,919 (74.2%)	Not available	14,358 (82.0%)
5·2- 6·1	5787 (14.3%)		2207 (12.6%)
≥6·2	2894 (7.2%)		812 (4.6%)
Not recorded	1734 (4.3%)		132 (0.8%)
**Total Cholesterol, mmol/L, mean(SD)**	4.4 (1.2)	4.6 (1.2)	4.2 (1.1)
**eGFR(ml/min/ 1·73m^2^**)			
<60	3592(8.9%)	9,309 (9·1%)	845 (4.8%)
≥60	28219(70.0%)	37,391 (36·4%)	16,664 (95.2%)
Not recorded	8523(21.1%)	55,972(54·5%)	0(0.0%)
**eGFR, ml/min/1.73m^2^, median(IQR)**	84.0 (71.0-90.0)	75.7 (63.1-89.1)	99.0 (83.9-113.4)
**Cardiovascular Disease**^b^			
Yes	5212 (12.9%)	24,571(23·9%)	771(4.4%)
**History of Antidiabetic medication**			
Diet-controlled	8925 (22.1%)	38,936 (37·9%)	224 (1.3%)
One drug	17,213 (42.7%)	36,900 (35·9%)	1955 (11.2%)
Two drugs	10,162 (25.2%)	22,617 (22·0%)	9757 (55.7%)
Insulin	4034 (10.0%)	4219 (4·1%)	5573 (31.8%)
**History of Antihypertensive medication (ever)**			
Yes	25,603 (63.5%)	77,221 (75·2%)	8454 (48.3%)
**History of background/mild or moderate retinopathy**			
Yes	8581 (21.3%)	20,417 (19·9%)	5418 (30.9%)

a Time since the diagnosis of diabetes.

b CVD includes Myocardial infarction, CHD, Atrial Fibrillation, Heart Failure and Stroke.

Supplement Table 4 shows the number of incident cases of STDR during the follow-up period and the incidence rate in model development and model validation datasets. The model development dataset had a total of 1427 people developed STDR events during the follow-up period, with an incidence rate per 1000 person years of 14.69 (95% CI: 13.94 to 15.47).

### Baseline characteristics of the study population

The highest proportion of people, 21,672 (53·7%) in model development and 57,352 (55·9%) in UK validation dataset had 0–2 years of duration of known diabetes. However, the highest proportion of people 7514 (42·9%) in Indian validation dataset had greater than 10 years of known duration of diabetes at baseline. Both the UK model development dataset and Indian model validation dataset had lower proportions of people aged 65+, 26% and 13% respectively compared with the UK model validation dataset with 48% of people aged 65 and above. More than 75% of people in UK were overweight or obese with a BMI≥25 kg/m^2^ whereas the Indian dataset had 67% people in the same group. HbA1c levels distribution was similar in UK model development dataset and Indian model validation dataset with around 13.2% people in the ≥ 80 mmol/mol, however the UK model validation dataset had 8.5% of people in this group. All three datasets had around 24%–37% people in highest SBP≥160 mmHg group. Total Cholesterol level was <5.2 mmol/L among 29,919 (77.5%) and 14,358 (82%) participants from UK model development and Indian model validation datasets respectively. More than 70% people in UK model development and Indian datasets had eGFR levels ≥ 60 ml/min/1.73 m^2^. There were 13% and 23% of people with history of CVD in UK development and validation datasets respectively, whereas the Indian model validation dataset had only 4% people with history of CVD. Around 80% and 60% of study participants were under at least one antidiabetic drug in the UK datasets and 98% in the Indian dataset respectively. History of antihypertensive medication was lowest among Indians 8454 (48%) compared with UK study participants in model development 25,603 (64%) and model validation 77,221 (75%) datasets. Highest proportion of people with background retinopathy 5,418 (31%) was reported among Indians whereas 21,417 (20%) and 8581 (21%) study participants in UK model validation and model development dataset had background retinopathy at baseline.

### Univariable analysis

Univariable results after considering all covariates that were pre-identified from literature have been presented in supplement Table 3. In univariable analysis, compared to those who are aged <45 years, people 65–74 years of age had higher risk of STDR (HR: 1·73; 95%CI 1·46 – 2·04) and those who were aged 75 and over had 38% higher risk of STDR (HR: 1.56; 95% CI 1·27–1.93). Females were at lower risk of STDR compared with males (HR: 0.91; 95%CI 0.82–1·01). South Asian and Black study participants had higher risk of STDR compared with White participants with 38% and 48% increased risk respectively. Those with HbA1c≥80 mmol/mol were almost seven times (HR:6·68; 95%CI 5·62–7·93) more likely to have STDR compared with those who have HbA1c<50 mmol/mol. Study participants with SBP≥160 mmHg were almost three times more likely to have STDR (HR: 2·55; 95% CI 2·05–3·18) compared to those who had SBP<120mmHg at baseline. People with eGfr≥60 ml/min/ 1.73m^2^ were 20% less likely to develop STDR (HR:0·79 95% CI 0·67–0·93) compared to those with eGFR<60 ml/min/1·73m^2^. There was significant increase in the risk of STDR by 60% among those with history of CVD (HR: 1·60; 95%CI 1.40–1·82) compared to those with no history of CVD. Study participants with history of insulin at baseline were 14 times more likely to have STDR compared to those who were not on any antidiabetic medication (HR: 14.14 95% CI 1.68–2.16). People with history of background retinopathy were 6.77 times more likely to develop STDR compared to those who did not have history of background retinopathy at baseline (HR: 6.77; 95% CI 6.08–7.54).

### Predictor variables, Predictive Models and their performance

Table 2 shows the final parsimonious model (Model 1) selected from backward elimination of predictor variables that were least significant and variables that did not reduce model performance significantly. The variables retained in the model were age at baseline, gender, duration of T2DM, history of antidiabetic medication, HbA1c level at baseline (± 6 months), and history of background retinopathy at baseline. Reduced model (Model 2) had all variables of Model 1 except history of background retinopathy which had the least contribution towards model performance. Further reduced model (Model 3) was a low-resource model with all non-clinical variables and included all variables in Model 2 except HbA1c.

**Table 2:** Hazard ratios for predictor variables in each development model predicting the three-year risk of STDR.

Characteristic	Model 1 (*N* = 40,334)	Model 2 (*N* = 40,334)	Model 3 (*N* = 40,334)
	Hazard Ratio (95% CI)	Hazard Ratio (95% CI)	Hazard Ratio (95% CI)
**Age**			
<45	1.00	1.00	1.00
45-54	1.17 (0.91-1.50)	1.21 (0.94-1.55)	1.16 (0.91-1.49)
55-64	1.16 (0.89-1.51)	1.25 (0.97-1.63)	1.11 (0.86-1.44)
65-74	1.65 (1.26-2.15)	1.70 (1.31-2.22)	1.44 (1.11-1.88)
75+	1.80 (1.27-2.53)	1.93 (1.38-2.70)	1.56 (1.12-2.19)
**Duration of Type 2 Diabetes (Years)**^a^	1.09 (1.06-1.11)	1.12 (1.09-1.15)	1.12 (1.10-1.15)
**Age by duration interaction**^a^^b^			
<45	1.00	1.00	1.00
45-54	0.99 (0.96-1.01)	0.98 (0.95-1.00)	0.98 (0.95-1.01)
55-64	0.97 (0.95-1.00)	0.96 (0.93-0.98)	0.96 (0.93-0.99)
65-74	0.96 (0.93-0.98)	0.94 (0.91-0.96)	0.94 (0.91-0.96)
75+	0.95 (0.92-0.97)	0.93 (0.90-0.95)	0.93 (0.90-0.95)
**Gender**			
Male	1.00	1.00	1.00
Female	0.89 (0.80-0.99)	0.84 (0.76-0.94)	0.83 (0.75-0.92)
**Antidiabetic History**			
Diet control	1.00	1.00	1.00
One drug	1.35 (1.05-1.73)	1.37 (1.07-1.75)	1.49 (1.16-1.90)
Two drugs	2.42 (1.91-3.07)	2.74 (2.16-3.47)	3.55 (2.81-4.48)
Insulin	3.43 (2.66-4.42)	4.45 (3.46-5.72)	6.75 (5.29-8.62)
**Hba1c (mmol/mol)**			
<50	1.00	1.00	
50-59	1.19 (0.98-1.44)	1.23 (1.02-1.49)	
60-69	1.69 (1.39-2.05)	1.80 (1.49-2.19)	
70-79	1.82 (1.47-2.25)	2.03 (1.64-2.50)	
80 and over	2.88 (2.39-3.46)	3.28 (2.73-3.93)	
**History of Background (mild or moderate) diabetic retinopathy**			
No	1.00		
Yes	3.71 (3.30-4.16)		

a Duration of type 2 diabetes was used a continuous variable in the model.

b Multiplicative modification to the duration slope for each age group.

### Internal validation and external validation

All three models had strong performance in discrimination with a C-statistic greater than 0.7 in the model development dataset as given in Table 3. Model performance in all ethnic groups of White, South Asians, Black and other were also strong with C-Statistic greater than 0.7 (Supplement Table 5). Model 1 and Model 2 had the highest performance among South Asians with a C-Statistic of 0·844 (95% CI 0·830–0·857) and 0.815 (95% CI 0.800–0.830) and Model 3 (non-invasive) model had slightly reduced model performance of 0·801 (95% CI 0·784–0·817). All three models had lowest performance among Black minority with C-Statistic ranging from 0·745 to 0·810.

**Table 3:** Model performance statistics in development and external validation datasets.

Performance Statistic	Development Dataset	External Validation	
		UK (Wales) Dataset	India – MDRF Dataset
**Model 1**			
C statistic (95% CI)	0·832 (0·822 to 0·842)	0.775 (0.767 to 0.783)	0.823 (0.810 to 0.836)
Calibration Slope^a^ (95% CI)	1·000	0.953 (0.924 to 0.982)	1.015 (0.939 to 1.091)
O/E Ratio	-	1.147	0.429
Re-calibrated O/E Ratio	-	1.017	1.026
**Model 2**			
C Statistic (95% CI)	0·795 (0·784 to 0·807)	0.707 (0.697 to 0.717)	0.742 (0.724 to 0.761)
Calibration Slope^a^ (95% CI)	1·000	0.853 (0.817 to 0.889)	0.921 (0.832 to 1.01)
O/E Ratio	-	1.221	0.429
Re-calibrated O/E Ratio	-	1.016	1.013
**Model 3 (Non-Invasive)**			
C Statistic (95% CI)	0·778 (0·766 to 0·790)	0.685 (0.675 to 0.695)	0.713 (0.693 to 0.733)
Calibration Slope^a^ (95% CI)	1·000	0.839 (0.799 to 0.879)	0.887 (0.786 to 0.987)
O/E Ratio	-	1.223	0.530
Re-calibrated O/E Ratio	-	1.021	1.018

a Beta-coefficient of the linear predictor, equal to 1 in model development dataset by definition. Shrunken (Heuristic) baseline survival at 3-years in model development dataset is 0.9947 for model 1, 0.9933 for model 2 and 0.9903 for model 3. Re-calibrated baseline survival for SAIL is 0.9940 for model 1, 0.9919 for model 2 and 0.9883 for model 3. Re-calibrated baseline survival for MDRF is 0.9980 for model 1, 0.9974 for model 2 and 0.9960 for model 3.

**Figure 1:**
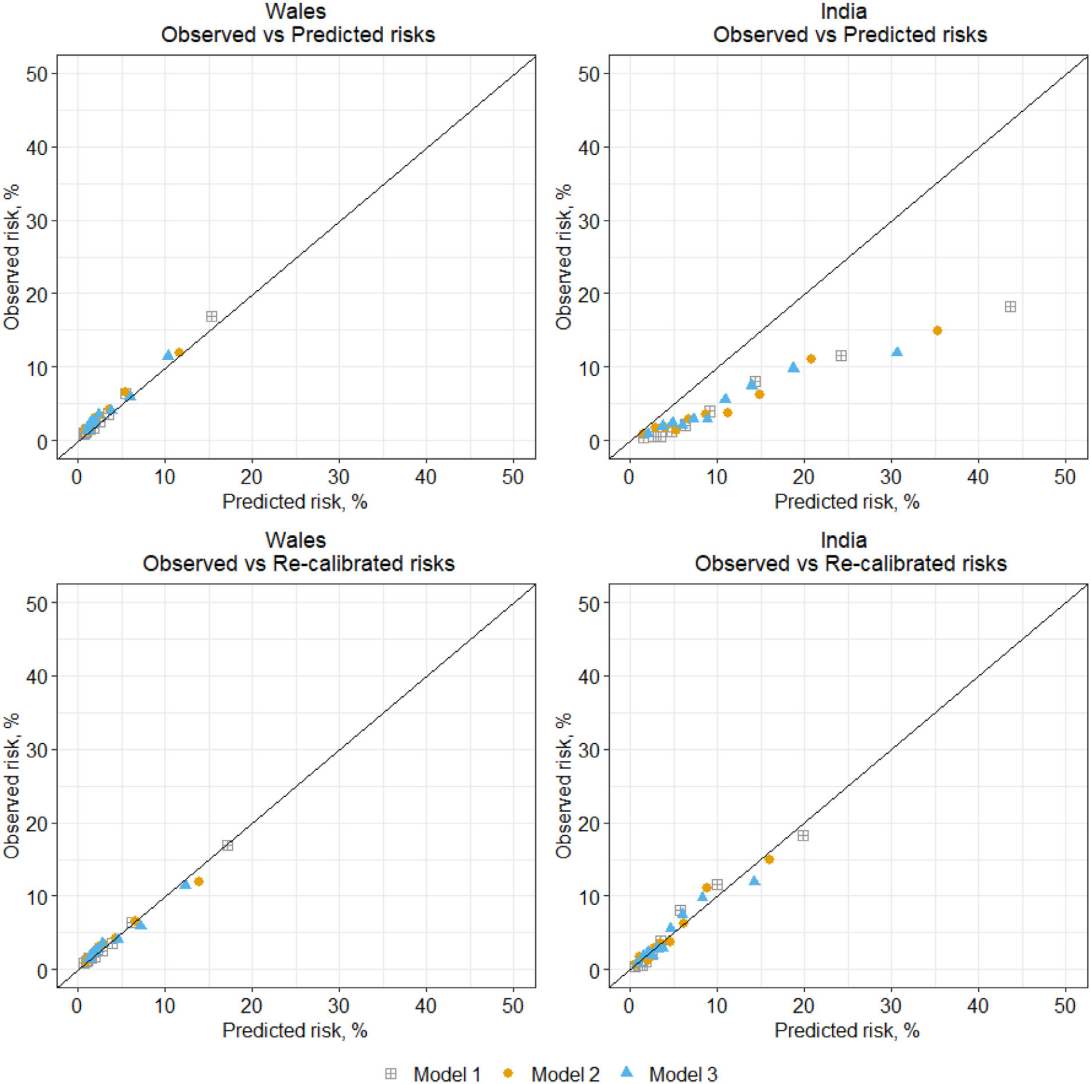
Observed and predicted 3-year risk of STDR event risk according to Model 1, Model 2, and Model 3 in validation^†^ ^†^Models mean predicted risks against observed risks (Kaplan-Meier event rates) for risk groups categorised into deciles of predicted risk for UK (Wales) and India datasets. Shrunken (Heuristic) baseline survival at 3-years in model development dataset is 0.9947 for model 1, 0.9933 for model 2 and 0.9903 for model 3. Re-calibrated baseline survival for SAIL is 0.9940 for model 1, 0.9919 for model 2 and 0.9883 for model 3. Re-calibrated baseline survival for MDRF is 0.9980 for model 1, 0.9974 for model 2 and 0.9960 for model 3.

In relation to the external validation, Model 1 had excellent performance both in the UK and Indian dataset with a C-statistic that is greater than 0.7 and calibration slope value closer to 1. Model 2 with fewer variables had slightly lower performance both in the UK and Indian datasets. Model 3 the non-invasive model had satisfactory performance in the UK and Indian dataset with C statistic of 0·685 and 0·713 respectively, and the observed to expected ratio closer to 1 following model re-calibration of the baseline survival function showed its applicability in both settings (Table 3). The beta coefficient of the linear predictor or calibration slope suggests that on average observed risks across all time points by 3 years were being over-estimated by our models in Wales and in India, except for model 1 in India which has a calibration slope > 1. The calibration plot in external validation datasets (Figure 1) was generated by categorising 3-year risks into 10 groups, and it suggest the predicted risks appear to be better aligned to the observed risks at 3-years in the UK (Wales) dataset than India. However, following re-calibration of the baseline survival, observed and predicted risks in both UK and India datasets were more closely aligned. Observed over expected (O/E) ratios show that all models applied in the Indian datasets, were on average over-predicting risks at 3 years and risks being slightly under-predicted in Wales at 3-years. However, re-calibrated O/E ratios for both UK (Wales) and India were all nearer to 1 (1.013-1·026).

### Model presentation

**Figure 2:**
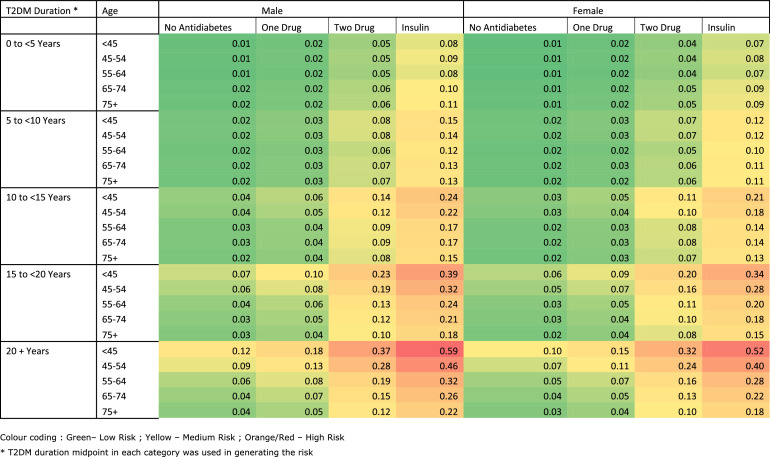
Risk-chart for 3-year risk of STDR using model 3 (Low-Resource Model) in the model development dataset.

The risk-chart (Figure 2) is a graphical representation of the risk score, and this shows the risk of each individual according to their group of T2DM duration, age, gender and antidiabetic medication. For example, a female aged 55 with one year duration of diabetes and on insulin has an estimated 3-year risk of 6% to develop STDR. Whereas a male aged 55 years with one year duration of diabetes and on insulin has an estimated 3-year risk of 8% to develop STDR. In addition, for those with duration more than 10 years the risk of STDR was peaking in the age group of <45 years, with further increased risk if they were taking two antidiabetic medications or be placed on insulin. Supplementary file Figure 1 and 2 provides the risk charts generated for UK validation dataset and Indian validation dataset.

### Sensitivity analysis

The incidence rates of STDR using the Turnbull's estimator which accounts for the interval censored nature of routine healthcare data, appear in close correspondence with Kaplan-Meier rates in the development cohort (Supplement Table 6). Similarly, Hazard ratios from the interval-censored Cox model presented in Supplement Table 7 are consistent with our final models generated using the Cox model assuming right censoring (Table 2).

## Discussion

We have developed and externally validated three risk prediction equations to estimate the absolute risk of STDR over a period of 3 years. The first model may be applied in settings where laboratory facilities and retinal screening is available. The second model is for settings where retinal screening is a challenge, but HbA1c is routinely available. The third model is developed for resource restricted settings. The equations are well calibrated and have good performance with C statistics close to 0.7 and above in both the development and external validation datasets.

To our knowledge this is the first study that has developed a risk prediction model for STDR to triage the at-risk group for retinal screening especially in resource restricted settings where systematic retinal screening is not available or accessible. Although, all people with diabetes should be screened regularly for STDR, nearly all low- and middle-income countries lack a systematic retinal screening programme and late presentation of STDR with irreversible visual loss is prevalent. The third model uses no laboratory markers or record of previous retinopathy status. Although, the least accurate of the three models, this model may be used by community workers to prioritise people at risk for retinal screening, facilitating efficient use of the limited capacity of retinal facilities. It may also be promoted as a self-assessment tool that can be used by people with diabetes to assess their risk of STDR. It may help them to make informed decisions about managing their level of diabetes and preventing or delaying STDR.

Undiagnosed diabetes also remains a global challenge. In our UK datasets, more than 50% of the people were newly diagnosed diabetes (≤2 years duration at baseline). However, some of them may present with STDR. Therefore, using these datasets enabled us to take this factor into account. Our risk models highlight that duration of diagnosed diabetes is by itself an insufficient predictor of STDR. Whilst several non-laboratory-based risk scores are available for community screening of diabetes, no similar models exist for identifying STDR alongside. On the contrary, a simple urine dipstick can identify albuminuria in all newly detected person with diabetes. Therefore, this model may also be useful in such circumstances.

Two recent systematic reviews on predictive models for diabetic retinopathy have summarised the existing literature on this topic and has identified several predictive models on severe diabetic retinopathy related outcomes including any form of retinopathy,^8^ blindness,^9^^,^^13^^,^^20^ diabetic macular oedema (DME) or proliferative diabetic retinopathy (PDR),^10^^,^^14^ treatment of DME/PDR,^12^ intraretinal microvascular abnormalities,^15^ STDR,^16^^,^^19^ retinopathy requiring photocoagulation,^21^ and other forms of severe diabetic retinopathy^23^^,^^24^ as presented in Supplement Table 1. These studies have used different data sources including routinely available databases such as The Health Improvement Network Data (THIN data)^18^ and Clinical Practice Research Datalink Data (CPRD),^20^ US claims database,^14^ hospital databases,^37^^,^^38^ diabetic eye screening data and clinical trials data.^9^^,^^15^ However, models utilising only non-laboratory parameters are required for application in low-resource settings.

Only a few of existing predictive models were identified with low risk of bias mainly because most had small sample sizes, missing data, or lacked external validation. Our study has filled this gap by introducing three models that could be chosen based on the resource setting. We also externally validated the models in two datasets from UK and India so that the models are tested in both high and middle-income countries. We have also provided a risk chart with a colour scheme to aid community workers. These models are not a replacement for retinal screening and should be used only for prioritisation for regular retinal screening.

One of the limitations of the study is that there were differences in the sources of our datasets. Our development dataset is a primary care dataset from London, where records are updated from the diabetic screening units while the SAIL de-identified dataset on the population of Wales obtain data from multiple resources including retinal screening episodes. The SAIL dataset had poor recording of ethnicity with (54,483) 53% missing values and by extension eGFR (which was calculated using the Modification of Diet in Renal Disease (MDRD) equation incorporating serum creatinine and ethnicity) with 54.5% missing values but none of these variables were included in the models and therefore did not have an impact on the results. However, further studies with complete data on all relevant covariates or studies using missing data analysis techniques such as multiple imputation to address missing values would be useful as part of any pilot or external validation of these models. The India dataset was from electronic medical records from an established diabetes centre. Furthermore, the English national diabetic eye screening classification of R2 also includes a proportion of people with less severe grades compared to the American Academy of Ophthalmology International Classification. In addition, there are inconsistencies in referral criteria used around the world and the use of “sight-threatening” and “vision-threatening” retinopathy. Therefore, further studies using alternative referral thresholds are required.

In conclusion, we have developed three predictive models to predict three-year risk of STDR that may be applied based on resource settings. These risk scores may be used to identify those who need prioritisation for retinal screening and treatment so that the rate of blindness due to STDR does not rise with the rising prevalence of diabetes. However, further testing and piloting of these models would be required, and they do not replace retinal screening but could be more useful as a pre-screening strategy until systematic retinal screening for all people with diabetes is made available globally.

## Contributors

SS and MN conceived the research question and designed the study with statistical input from ATP. SS and MN had full access and verified all the data in the study and take responsibility for the integrity of the data and the accuracy of the data analysis. MN, SG, RM, JR, SJ, JMR collected the data, and MN and SG conducted the analysis under supervision of ATP and SS. ATP, SS, RR, TS, MA, VM, DO supported drafting the manuscript. All authors critically revised the manuscript for important intellectual content and gave final approval for the version to be published. All authors agree to be accountable for all aspects of the work in ensuring that questions related to the accuracy or integrity.

## Data sharing statement

Restrictions apply to the availability of this data. This study was conducted using de-identified data from general practice electronic health records collected in three Clinical Commissioning Groups (CCGs) in East London and data are available with permission from the Caldicott guardian of this anonymised dataset in Queen Mary University London (QMUL).

## Declaration of interests

SS reports personal fees from Novartis, personal fees from Bayer, grants from Boehringer Ingleheim, grants and personal fees from Allergan, personal fees from Oxurion, personal fees from Apellis, personal fees from Roche, outside the submitted work; ATP reports grants from UKRI to employer King's College London, during the conduct of the study; personal fees from Roche and a grant from Wellcome Trust, outside the submitted work. RM reports personal fees from AMGEN, outside the submitted work. None of the other authors declare that they have any competing interests related to the submitted work.

## Appendix Supplementary materials

Image, application 1
