# Supplementary material for: Development and validation of predictive risk models for sight threatening diabetic retinopathy in patients with type 2 diabetes to be applied as triage tools in resource limited settings
Source: eClinicalMedicine. 2022 Jul 22;51:101578. doi: 10.1016/j.eclinm.2022.101578 (PMC9310126; doi:10.1016/j.eclinm.2022.101578)
Supplement: Supplementary file 1 [file mmc1.docx]

Supplementary File

Supplement Figure 1: Flowchart of inclusion and exclusion of cohort participants (model development dataset)

Supplement Table 1: Existing Models on DR, STDR and Blindness prediction and limitations for use in low-resource settings

Supplement Table 2: Predictors in the existing models for DR/STDR and blindness

Supplement Table 3: Model Development Dataset – Univariable analysis of predictors with the outcome

Supplement Table 4: Number of incident cases of STDR during 3-year follow-up and incidence rates per 1000 person years in each dataset

Supplement Table 5: Model performance statistics for different ethnic groups in the model development dataset - QMUL (London) dataset

Supplement Table 6: Comparing incidence rates between Kaplan-Meier and Turnbull’s estimator in model development dataset – QMUL (London) dataset

Supplement Table 7: Sensitivity analysis (interval censored cox models) Hazard ratios for model development – QMUL (London) dataset

Supplement Figure 2: Risk Chart for 3-year risk of STDR using Model 3 (Non-Invasive Model) in UK validation dataset – SAIL Data

Supplement Figure 3: Risk Chart for 3-year risk of STDR using Model 3 (Non-Invasive Model) in Indian validation dataset

Supplement Figure 1: Flowchart of inclusion and exclusion of cohort participants (model development dataset)

Patients identified with any clinical code for T2DM and screened for DR in the period 2007-2017 (n=71,908)

Records excluded (n=25,384)

- Those who died before study start (n=3)
- Missing dob (n=1)
- Those who left the practice before study start (n=2,059)
- Those who registered on or after study end (n=43)
- Those who turned 18 on or after study end (n=31)
- Enter study>=exit study (n=30)
- Those who were diagnosed with diabetes before the age of 18 (n=186)
- Those diagnosed after exit study (n=0)
- Those without DR screening date or a DR diagnosis date between cohort entry and exit (n=5,028)
- Recording errors: T2DM onset after first screening appointment during entry and exit dates (n=1,274)
- On insulin on or prior to T2DM onset (n=1,497)
- On insulin prior to 30^th^ birthday (n=402)
- On any anti-diabetic medication prior to T2DM onset (n=4,878)
- Do not have a [(a second screening) OR (recorded DR event during the study period) OR (a screening event or DR event after study end more than 6 months gap prior to STDR onset) OR (a screening event or BGDR event after study end for those with no STDR onset)] (n=9,952)

T2DM patients eligible for the study, with complete **demographic** data (n=46,524)

Record excluded (n=6,190)

- Prior SDTR diagnosis or within 6 months from baseline date (n=2,946)
- Those without hba1c measured within 6 months from baseline date (n=3,244)

T2DM patients free from STDR at baseline, with complete covariate data (n=40,334)

Supplement Table 1: Existing Models on DR, STDR and Blindness prediction and limitations for use in low-resource settings

| Model Number | Reference | Setting | Population | Sample Size | Events | Prediction period | Outcome | Limitations in low-resource settings |
| --- | --- | --- | --- | --- | --- | --- | --- | --- |
| 1 | Aspinall et al,^1^  1983 | Scotland | T1DM and  T2DM | 295 | 86 | 7 years | Any form of retinopathy | Include T1DM, colour vision, require 6 previous post-prandial blood sugar estimation and proteinuria |
| 2 | Clarke et al,^2^  2004 | UK | T2DM | 3,642 | NR | Lifetime | Blindness | Simulation Model |
| 3 | Aspelund et al,^3^ 2011 | Iceland, UK, USA | T1DM and T2DM | T1DM: 634  T2DM: 929 | T1DM: 235  T2DM: 61 | 5 years | DME or PDR | Include T1DM, Model needs HbA1c and previous record of DR status |
| 4 | Semeraro et al,^4^ 2011 | Italy | T2DM | 5034 | 569 | 1,2,3,4 years | DR | Outcome is DR· Predictors include HbA1c, albuminuria and creatinine clearance· |
| 5 | Mehlsen et al,^5^ 2012 | Denmark | T1DM  T2DM | T1DM: 1,275  T2DM: 3,572 | T1DM: 171  T2DM: 388 | 3 years | Treatment of DME/PDR | Includes T1DM; Predictors include retinal haemorrhages, exudates and HbA1c |
| 6 | Hayes,^6^  2013 | UK | T2DM | 5,102 | 271 | Lifetime | Blindness | Requires HbA1c, systolic blood pressure, heart rate and white blood cells count |
| 7 | Harris,^7^  2013 | US | NR | 4,617 | 307 | 3, 5 years | Proliferative Diabetic Retinopathy | Requires HbA1c and data on presence of diabetic nephropathy |
| 8 | Tanaka et al,^8^  2013 | Japan | T2DM | 1,748 | 415 | 5 years | Intraretinal microvascular abnormalities | Outcome is only 1 retinal finding· Predictors include HbA1c and albumin: creatinine ratio |
| 9 | Stratton,^9^  2013 | UK | T1DM  T2DM | 14,554 | 499 | NR | Sight threatening DR | Requires data on presence of retinopathy |
| 10 | Lagani,^10^  2015 | UK | T1DM  T2DM | 49  344 | 17  70 | 6·5 years | Retinopathy | Requires HbA1c and data on presence of retinopathy |
| 11 | McEwan,^11^  2015 | UK | T2DM | 123,159 | 1177 | 25 years | Blindness | Requires HbA1c, SBP and LDL |
| 12 | Scanlon et al,^12^ 2015 | UK | T1DM  T2DM | 7,012 | 606 | 5 years | Referable DR and maculopathy | Includes T1DM; Require HbA1c, creatinine, total cholesterol and retinopathy status |
| 13 | Hippisley-Cox and Coupland,^13^ (2015) | UK | T1DM  T2DM | 454,575 | 8,063 | 10 years | Blindness | Includes T1DM, Require HbA1c, Cholesterol: HDL ratio and DR status |
| 14 | Basu et al,^14^ (2017) | USA/Canada | T2DM | 9,635 | 901 | 10 years | Retinopathy requiring photocoagulation | Require HbA1c, HDL, cholesterol, creatinine, history of CVD |
| 15 | Eleuteri et al,^15^  2017 | UK | T1DM  T2DM | 11,806 | 388 | 6, 12, 24 months | Sight threatening diabetic retinopathy | Requires HbA1c, Cholesterol and SBP |
| 16 | Dagliati et al,^16^ 2018 | Italy | T2DM | 943 | 118 | 3, 5, 7 years | Specific lesions at dilated fundoscopy | Outcome is not specific for STDR; require HbA1c |
| 17 | Gracia-Finana et al,^17^ 2019 | UK | T1DM  T2DM | T1DM: 651  T2DM:  12, 452 | T1DM: 66  T2DM: 275 | 1 year | Moderate and severe DR | Includes T1DM; require HbA1c, DR status, previous screening attendance |
| 18 | Ochs et al,^18^  2019 | UK | T1DM  T2DM | T1DM: 19,070  T2DM:  220,276 |  | 5 years | Referable DR | Includes T1DM; require HbA1c, cholesterol and previous DR status· |

Abbreviations: T1DM-Type one Diabetes Mellitus, T2DM- Type two Diabetes Mellitus

Supplement Table 2: Predictors in the existing models for DR/STDR and blindness

| **Predictor** | Aspinall et al, 1983 | Clarke et al, 2004 | Aspelund et al, 2011 | Semeraro et al, 2011 | Mehlsen et al, 2012 | Hayes, 2013 | Harris, 2013 | Tanaka et al, 2013 | Stratton, 2013 | Lagani, 2015 | Scanlon et al, 2015 | Hippisely-Cox, 2015 | Basu et al, 2017 | Dagliati et al, 2018 | Garcia-Finana et al, 2019 | Ochs et al, 2019 |
| --- | --- | --- | --- | --- | --- | --- | --- | --- | --- | --- | --- | --- | --- | --- | --- | --- |
| **Sociodemographic Characteristics** |  |  |  |  |  |  |  |  |  |  |  |  |  |  |  |  |
| Age |  | **×** |  |  |  | **×** |  | **×** | **×** |  |  |  | **×** |  |  | **×** |
| Gender |  |  | **×** | **×** | **×** |  |  |  | **×** |  |  | **×** | **×** |  |  |  |
| Deprivation |  |  |  |  |  |  |  |  |  |  |  |  | **×** |  |  |  |
| Marital Status |  |  |  |  |  |  |  |  |  | **×** |  |  |  |  |  |  |
| Ethnicity |  |  |  |  |  |  |  |  |  |  |  |  | **×** |  |  |  |
| Smoking Status |  |  |  |  |  |  |  |  |  |  |  |  |  | **×** |  |  |
| BMI |  |  |  |  |  |  |  | **×** |  | **×** |  |  |  | **×** |  |  |
| Age at diagnosis |  |  |  |  | **×** |  |  |  |  |  |  |  |  |  |  |  |
| Diabetes duration | **×** |  | **×** | **×** | **×** |  |  | **×** |  |  | **×** | **×** |  |  | **×** | **×** |
| Type of diabetes |  |  | **×** |  |  |  |  |  |  |  |  | **×** |  |  | **×** |  |
| Postprandial blood glucose | **×** |  |  |  |  |  |  |  |  |  |  |  |  |  |  |  |
| **Clinical** |  |  |  |  |  |  |  |  |  |  |  |  |  |  |  |  |
| HbA1c |  | **×** | **×** | **×** | **×** | **×** | **×** | **×** |  | **×** | **×** |  | **×** | **×** | **×** | **×** |
| **Predictor** | Aspinall et al, 1983 | Clarke et al, 2004 | Aspelund et al, 2011 | Semeraro et al, 2011 | Mehlsen et al, 2012 | Hayes, 2013 | Harris, 2013 | Tanaka et al, 2013 | Stratton, 2013 | Lagani, 2015 | Scanlon et al, 2015 | Hippisely-Cox, 2015 | Basu et al, 2017 | Dagliati et al, 2018 | Garcia-Finana et al, 2019 | Ochs et al, 2019 |
| Systolic Blood pressure |  |  | **×** | **×** |  | **×** |  |  |  |  |  |  | **×** |  | **×** |  |
| Total cholesterol |  |  |  |  |  |  |  |  |  |  | **×** |  | **×** |  |  | **×** |
| HDL |  |  |  |  |  |  |  |  |  |  |  |  | **×** |  |  |  |
| Cholesterol/HDL ratio |  |  |  |  |  |  |  |  |  |  |  | **×** |  |  |  |  |
| Serum creatinine |  |  |  |  |  |  |  |  |  |  | **×** |  | **×** |  |  |  |
| Albumin-creatinine ratio |  |  |  |  |  |  |  | **×** |  |  |  |  | **×** |  |  |  |
| Albuminuria |  |  |  | **×** |  |  |  |  |  |  |  |  |  |  |  |  |
| Creatinine clearance |  |  |  | **×** |  |  |  |  |  |  |  |  |  |  |  |  |
| **Comorbidities** |  |  |  |  |  |  |  |  |  |  |  |  |  |  |  |  |
| History of CVD |  |  |  |  |  | **×** |  |  |  |  |  |  | **×** |  |  |  |
| Chronic renal disease |  |  |  |  |  |  |  |  |  |  |  | **×** |  |  |  |  |
| Proteinuria | **×** |  |  |  |  |  |  |  |  |  |  |  |  |  |  |  |
| Diabetic nephropathy |  |  |  |  |  |  | **×** |  |  |  |  |  |  |  |  |  |
| Nonhealing ulcers |  |  |  |  |  |  | **×** |  |  |  |  |  |  |  |  |  |
| **Medication use** |  |  |  |  |  |  |  |  |  |  |  |  |  |  |  |  |
| Antihypertenstive |  |  |  |  |  |  |  |  |  |  |  |  | **×** | **×** |  |  |
| Antidiabetic drugs |  |  |  |  |  |  |  |  |  |  |  |  | **×** |  |  |  |
| **Predictor** | Aspinall et al, 1983 | Clarke et al, 2004 | Aspelund et al, 2011 | Semeraro et al, 2011 | Mehlsen et al, 2012 | Hayes, 2013 | Harris, 2013 | Tanaka et al, 2013 | Stratton, 2013 | Lagani, 2015 | Scanlon et al, 2015 | Hippisely-Cox, 2015 | Basu et al, 2017 | Dagliati et al, 2018 | Garcia-Finana et al, 2019 | Ochs et al, 2019 |
|  |  |  |  |  |  |  |  |  |  |  |  |  |  |  |  |  |
| **Eye exam** |  |  |  |  |  |  |  |  |  |  |  |  |  |  |  |  |
| Presence of retinopathy |  |  | **×** | **×** |  |  |  |  | **×** | **×** | **×** | **×** |  |  | **×** | **×** |
| Colour discrimination | **×** |  |  |  |  |  |  |  |  |  |  |  |  |  |  |  |
| Hard exudates |  |  |  |  | **×** |  |  |  |  |  |  |  |  |  |  |  |
| Haemorrhages |  |  |  |  | **×** |  |  |  |  |  |  |  |  |  |  |  |
| Previous appointment |  |  |  |  |  |  |  |  |  |  |  |  |  |  | **×** |  |
| **Others** |  |  |  |  |  |  |  |  |  |  |  |  |  |  |  |  |
| Heart rate |  |  |  |  |  | **×** |  |  |  |  |  |  |  |  |  |  |
| White blood cells |  |  |  |  |  | **×** |  |  |  |  |  |  |  |  |  |  |
| Post pubescent diabetes duration |  |  |  |  |  |  |  |  |  | **×** |  |  |  |  |  |  |

Supplement Table 3: Model Development Dataset – Univariable analysis of predictors with the outcome

|  | Hazard Ratio - unadjusted | P value |
| --- | --- | --- |
| **Duration of diabetes at baseline, years** | 1.10(1.10-1.11) | <.001 |
| **Age, years**  <45  45-54  55-64  65-74  75+ | Ref  1.38(1.17-1.62)  1.35(1.14-1.60)  1.73(1.46-2.04)  1.56(1.27-1.93) | -  <.001  <.001  <.001  <.001 |
| **Gender**  Male  Female | Ref  0.91(0.82-1.01) | -  .083 |
| **Ethnicity**  White  South Asian  Black  Other  Not recorded | Ref  1.38(1.20-1.58)  1.48(1.26-1.73)  1.41(1.11-1.79)  1.53(0.88-2.67) | -  <.001  <.001  .005  .134 |
| **Body Mass Index (kg/m^2^)***  <18·5  18·5 – 25  25-30  ≥30  Not recorded | Ref  1.09(0.66-1.81)  0.86(0.52-1.41)  0.75(0.46-1.24)  2.49(1.50-4.14) | -  .736  .540  .261  <.001 |
| **HbA1c ***  <50  >=50 & <60  >=60 & <70  >=70 & <80  >=80 | Ref  1.57(1.30-1.89)  3.27(2.71-3.94)  4.22(3.44-5.17)  6.68(5.62-7.93) | -  <.001  <.001  <.001  <.001 |
| **Systolic Blood Pressure – SBP***  <120  120-129  130-140  140-150  150-160  ≥160  Not recorded | Ref  1.19(1.00-1.42)  1.28(1.08-1.52)  1.77(1.48-2.11)  2.37(1.91-2.95)  2.55(2.05-3.18)  4.70(3.53-6.27) | -  .051  .004  <.001  <.001  <.001  <.001 |
| **Total Cholesterol ***(mmol/L)  <5·2  5·2- 6·1  ≥6·2  Not recorded | Ref  0.83(0.71-0.98)  0.92(0.74-1.15)  2.49(2.09-2.97) | -  .032  .480  <.001 |
| **eGFR***  <60  ≥60  Not recorded | Ref  0.50(0.43-0.58)  0.79(0.67-0.93) | -  <.001  .005 |
| **Cardiovascular Disease History†**  No  Yes | Ref  1.60(1.40-1.82) | -  <.001 |
| **Antidiabetic Drugs History**  No drug  One drug  Two drugs  Insulin | Ref  1.47(1.15-1.88)  5.24(4.17-6.58)  14.14(11.25-17.76) | -  .002  <.001  <.001 |
| **Antihypertensive drugs** History – ever  No  Yes | Ref  1.90(1.68-2.16) | -  <.001 |
| **History of background retinopathy**  No  Yes | Ref  6.77(6.08-7.54) | -  <.001 |

**recorded within 6 moths from baseline*

†CVD includes Myocardial infarction, CHD, Atrial Fibrillation, Heart Failure and Stroke

Supplement Table 4: Number of incident cases of STDR during 3-year follow-up and incidence rates per 1000 person years in each dataset

| **Ethnicity** | Total number of people | **STDR** | | | |
| --- | --- | --- | --- | --- | --- |
|  |  | Number of events | Percentage with events | Person-years | Incidence Rate per 1000 person years (95% CI) |
| UK – Model Development dataset | 40,334 | 1,427 | 3·54% | 97,172·30 | 14·69 (13·94 – 15·47) |
| UK – Model Validation Dataset | 102,672 | 3,337 | 3·25% | 269,455·10 | 12·38 (11·97 – 12·81) |
| India – Model Validation Dataset | 17,509 | 606 | 3·46% | 40,992·90 | 14·78 (13·65 - 16·01) |

Supplement Table 5: Model performance statistics for different ethnic groups in the model development dataset - QMUL(London) dataset

| Performance Statistic | White (N=10,572) | South Asian (N=26,357) | Black (N=11,827) | Other (N=3,343) |
| --- | --- | --- | --- | --- |
| **Model 1** |  |  |  |  |
| C statistic | 0.825(0.801-0.849) | 0.844(0.830-0.857) | 0.810(0.787-0.832) | 0.836(0.782-0.889) |
| Calibration Slope | 0.980(0.893-1.067) | 1.042(0.982-1.101) | 0.919(0.834-1.005) | 1.072(0.873-1.270) |
| **Model 2** |  |  |  |  |
| C Statistic | 0.768(0.741-0.794) | 0.815(0.800-0.830) | 0.776(0.752-0.801) | 0.796(0.744-0.849) |
| Calibration Slope* | 0.929(0.829-1.029) | 1.058(0.993-1.123) | 0.927(0.833-1.022) | 0.996(0.782-1.210) |
| **Model 3 (Non-Invasive)** |  |  |  |  |
| C Statistic | 0.761(0.734-0.788) | 0.801(0.784-0.817) | 0.745(0.717-0.772) | 0.774(0.716-0.831) |
| Calibration Slope* | 0.955(0.850-1.061) | 1.071(1.002-1.140) | 0.881(0.779-0.982) | 0.956(0.742-1.169) |

Supplement Table 6: Comparing incidence rates between Kaplan-Meier and Turnbull’s estimator in model development dataset – QMUL (London) dataset

| Follow-up time since baseline, years | 1 | 2 | 3 |
| --- | --- | --- | --- |
| Incidence of STDR using Kaplan-Meier method | 0.01 | 0.026 | 0.046 |
| Incidence of STDR using Turnbull's estimator | 0.02 | 0.033 | 0.042 |

Turnbull’s estimator generated using the R package *Interval^19^*

Supplement Table 7: Sensitivity analysis (interval censored cox models) Hazard ratios for model development – QMUL (London) dataset

| **Characteristic** | **Model 1**  **(N=40,334)** | | | | **Model 2**  **(N=40,334)** | | | | **Model 3**  **(N=40,334)** | | | |
| --- | --- | --- | --- | --- | --- | --- | --- | --- | --- | --- | --- | --- |
|  | HR | Log-HR | SE | P-value | HR | Log-HR | SE | P-value | HR | Log-HR | SE | P-value |
| **Age**  <45  45-54  55-64  65-74  75+ | Ref  1.19  1.17  1.69  1.79 | -  0.17  0.16  0.53  0.58 | -  0.12  0.14  0.14  0.17 | -  .154  .250  <.001  <.001 | Ref  1.23  1.27  1.75  1.92 | -  0.21  0.24  0.56  0.65 | -  0.12  0.13  0.13  0.17 | -  .077  .565  <.001  <.001 | Ref  1.18  1.13  1.49  1.55 | -  0.17  0.12  0.40  0.44 | -  0.12  0.14  0.13  0.17 | -  .165  .374  .003  .009 |
| **Duration of Type 2 Diabetes (Years)**^a^ | 1.09 | 0.08 | 0.01 | <.001 | 1.12 | 0.11 | 0.01 | <.001 | 1.12 | 0.12 | 0.01 | <.001 |
| **Age by duration interaction** ^a b^  <45  45-54  55-64  65-74  75+ | Ref  0.99  0.97  0.96  0.95 | -  -0.01  -0.03  -0.05  -0.06 | -  0.01  0.01  0.01  0.01 | -  .362  .069  .002  <.001 | Ref  0.98  0.96  0.94  0.93 | -  -0.02  -0.04  -0.06  -0.08 | -  0.01  0.01  0.01  0.01 | -  .088  .001  <.001  <.001 | Ref  0.98  0.96  0.94  0.93 | -0.02  -0.04  -0.06  -0.08 | -  0.01  0.01  0.01  0.01 | -  .010  .002  <.001  <.001 |
| **Gender**  Male  Female | Ref  0.90 | -  0.11 | -  0.05 | -  .047 | Ref  0.85 | -  0.16 | -  0.05 | -  .003 | Ref  0.84 | -  -0.17 | -  0.05 | -  <.001 |
| **Antidiabetic History**  Diet control  One drug  Two drugs  Insulin | Ref  1.36  2.47  3.53 | -  0.30  0.90  1.26 | -  0.13  0.12  0.14 | -  .018  <.001  <.001 | Ref  1.38  2.80  4.56 | -  0.32  1.03  1.52 | -  0.12  0.13  0.13 | -  .008  <.001  <.001 | Ref  1.50  3.64  6.95 | -  0.40  1.29  1.94 | -  0.13  0.13  0.14 | -  .002  <.001  <.001 |
| **Hba1c**  <50  50-59  60-69  70-79  80 and over | Ref  1.21  1.71  1.84  2.88 | -  0.19  0.54  0.61  1.06 | -  0.10  0.10  0.11  0.10 | -  .053  <.001  <.001  <.001 | Ref  1.25  1.83  2.05  3.27 | -  0.22  0.60  0.72  1.19 | -  0.09  0.09  0.10  0.09 | -  .01  <.001  <.001  <.001 |  |  |  |  |
| **History of Background (mild or moderate) diabetic retinopathy**  No  Yes | Ref  3.68 | -  1.30 | -  0.06 | -  <.001 |  |  |  |  |  |  |  |  |

Models were generated using the R package IcenReg, 500 bootstrap replicates were used in generating the standard error of the log-HR’s^20^

Supplement Figure 2: Risk Chart for 3-year risk of STDR using Model 3 (Non-Invasive Model) in UK validation dataset – SAIL Data

| T2DM Duration | Age |  | Male |  |  |  | Female |  |  |
| --- | --- | --- | --- | --- | --- | --- | --- | --- | --- |
|  |  | No Antidiabetes | One Drug | Two Drug | Insulin | No Antidiabetes | One Drug | Two Drug | Insulin |
| 0 to <5 Years | <45 | 0.02 | 0.02 | 0.05 | 0.10 | 0.01 | 0.02 | 0.05 | 0.08 |
|  | 45-54 | 0.02 | 0.03 | 0.06 | 0.11 | 0.01 | 0.02 | 0.05 | 0.09 |
|  | 55-64 | 0.02 | 0.02 | 0.05 | 0.10 | 0.01 | 0.02 | 0.05 | 0.08 |
|  | 65-74 | 0.02 | 0.03 | 0.07 | 0.12 | 0.02 | 0.02 | 0.06 | 0.10 |
|  | 75+ | 0.02 | 0.03 | 0.07 | 0.13 | 0.02 | 0.02 | 0.06 | 0.11 |
| 5 to <10 Years | <45 | 0.03 | 0.04 | 0.09 | 0.17 | 0.02 | 0.03 | 0.08 | 0.15 |
|  | 45-54 | 0.03 | 0.04 | 0.09 | 0.17 | 0.02 | 0.03 | 0.08 | 0.14 |
|  | 55-64 | 0.02 | 0.03 | 0.08 | 0.14 | 0.02 | 0.03 | 0.07 | 0.12 |
|  | 65-74 | 0.02 | 0.04 | 0.09 | 0.16 | 0.02 | 0.03 | 0.07 | 0.13 |
|  | 75+ | 0.02 | 0.04 | 0.08 | 0.15 | 0.02 | 0.03 | 0.07 | 0.13 |
| 10 to <15 Years | <45 | 0.05 | 0.07 | 0.16 | 0.29 | 0.04 | 0.06 | 0.14 | 0.24 |
|  | 45-54 | 0.04 | 0.06 | 0.14 | 0.26 | 0.04 | 0.05 | 0.12 | 0.22 |
|  | 55-64 | 0.03 | 0.05 | 0.11 | 0.20 | 0.03 | 0.04 | 0.09 | 0.17 |
|  | 65-74 | 0.03 | 0.05 | 0.11 | 0.20 | 0.03 | 0.04 | 0.09 | 0.17 |
|  | 75+ | 0.03 | 0.04 | 0.10 | 0.18 | 0.02 | 0.04 | 0.08 | 0.15 |
| 15 to <20 Years | <45 | 0.09 | 0.12 | 0.27 | 0.45 | 0.07 | 0.10 | 0.23 | 0.39 |
|  | 45-54 | 0.07 | 0.10 | 0.22 | 0.38 | 0.06 | 0.08 | 0.19 | 0.32 |
|  | 55-64 | 0.05 | 0.07 | 0.16 | 0.28 | 0.04 | 0.06 | 0.13 | 0.24 |
|  | 65-74 | 0.04 | 0.06 | 0.14 | 0.25 | 0.03 | 0.05 | 0.12 | 0.21 |
|  | 75+ | 0.04 | 0.05 | 0.12 | 0.21 | 0.03 | 0.04 | 0.10 | 0.18 |
| 20 + Years | <45 | 0.15 | 0.21 | 0.43 | 0.66 | 0.12 | 0.18 | 0.37 | 0.59 |
|  | 45-54 | 0.11 | 0.15 | 0.33 | 0.53 | 0.09 | 0.13 | 0.28 | 0.46 |
|  | 55-64 | 0.07 | 0.10 | 0.22 | 0.38 | 0.06 | 0.08 | 0.19 | 0.32 |
|  | 65-74 | 0.05 | 0.08 | 0.18 | 0.31 | 0.04 | 0.07 | 0.15 | 0.26 |
|  | 75+ | 0.04 | 0.06 | 0.14 | 0.25 | 0.04 | 0.05 | 0.12 | 0.22 |

Colour coding : Green– Low Risk ; Yellow – Medium Risk ; Orange/Red – High Risk

* T2DM duration midpoint in each category was used in generating the risk

Supplement Figure 3: Risk Chart for 3-year risk of STDR using Model 3 (Non-Invasive Model) in **Indian validation dataset**

| T2DM Duration | Age |  | Male |  |  |  | Female |  |  |
| --- | --- | --- | --- | --- | --- | --- | --- | --- | --- |
|  |  | No Antidiabetes | One Drug | Two Drug | Insulin | No Antidiabetes | One Drug | Two Drug | Insulin |
| 0 to <5 Years | <45 | 0.01 | 0.01 | 0.02 | 0.04 | 0.00 | 0.01 | 0.02 | 0.03 |
|  | 45-54 | 0.01 | 0.01 | 0.02 | 0.04 | 0.00 | 0.01 | 0.02 | 0.03 |
|  | 55-64 | 0.01 | 0.01 | 0.02 | 0.04 | 0.00 | 0.01 | 0.02 | 0.03 |
|  | 65-74 | 0.01 | 0.01 | 0.02 | 0.04 | 0.01 | 0.01 | 0.02 | 0.04 |
|  | 75+ | 0.01 | 0.01 | 0.02 | 0.05 | 0.01 | 0.01 | 0.02 | 0.04 |
| 5 to <10 Years | <45 | 0.01 | 0.01 | 0.03 | 0.06 | 0.01 | 0.01 | 0.03 | 0.05 |
|  | 45-54 | 0.01 | 0.01 | 0.03 | 0.06 | 0.01 | 0.01 | 0.03 | 0.05 |
|  | 55-64 | 0.01 | 0.01 | 0.03 | 0.05 | 0.01 | 0.01 | 0.02 | 0.04 |
|  | 65-74 | 0.01 | 0.01 | 0.03 | 0.06 | 0.01 | 0.01 | 0.03 | 0.05 |
|  | 75+ | 0.01 | 0.01 | 0.03 | 0.06 | 0.01 | 0.01 | 0.02 | 0.05 |
| 10 to <15 Years | <45 | 0.02 | 0.03 | 0.06 | 0.11 | 0.01 | 0.02 | 0.05 | 0.09 |
|  | 45-54 | 0.01 | 0.02 | 0.05 | 0.10 | 0.01 | 0.02 | 0.04 | 0.08 |
|  | 55-64 | 0.01 | 0.02 | 0.04 | 0.07 | 0.01 | 0.01 | 0.03 | 0.06 |
|  | 65-74 | 0.01 | 0.02 | 0.04 | 0.07 | 0.01 | 0.01 | 0.03 | 0.06 |
|  | 75+ | 0.01 | 0.02 | 0.04 | 0.07 | 0.01 | 0.01 | 0.03 | 0.06 |
| 15 to <20 Years | <45 | 0.03 | 0.04 | 0.10 | 0.19 | 0.03 | 0.04 | 0.09 | 0.16 |
|  | 45-54 | 0.02 | 0.04 | 0.08 | 0.15 | 0.02 | 0.03 | 0.07 | 0.13 |
|  | 55-64 | 0.02 | 0.02 | 0.06 | 0.11 | 0.01 | 0.02 | 0.05 | 0.09 |
|  | 65-74 | 0.01 | 0.02 | 0.05 | 0.09 | 0.01 | 0.02 | 0.04 | 0.08 |
|  | 75+ | 0.01 | 0.02 | 0.04 | 0.08 | 0.01 | 0.02 | 0.04 | 0.07 |
| 20 + Years | <45 | 0.05 | 0.08 | 0.18 | 0.31 | 0.04 | 0.07 | 0.15 | 0.26 |
|  | 45-54 | 0.04 | 0.06 | 0.13 | 0.23 | 0.03 | 0.05 | 0.11 | 0.19 |
|  | 55-64 | 0.02 | 0.04 | 0.08 | 0.15 | 0.02 | 0.03 | 0.07 | 0.13 |
|  | 65-74 | 0.02 | 0.03 | 0.06 | 0.12 | 0.02 | 0.02 | 0.05 | 0.10 |
|  | 75+ | 0.01 | 0.02 | 0.05 | 0.10 | 0.01 | 0.02 | 0.04 | 0.08 |

Colour coding : Green– Low Risk ; Yellow– Medium Risk ; Orange/Red – High Risk

* T2DM duration midpoint in each category was used in generating the risk

# References:

1. Aspinall PA, Kinnear PR, Duncan LJ, Clarke BF. Prediction of diabetic retinopathy from clinical variables and color vision data. *Diabetes Care* 1983; **6**(2): 144-8.

2. Clarke PM, Gray AM, Briggs A, et al. A model to estimate the lifetime health outcomes of patients with type 2 diabetes: the United Kingdom Prospective Diabetes Study (UKPDS) Outcomes Model (UKPDS no. 68). *Diabetologia* 2004; **47**(10): 1747-59.

3. Aspelund T, Þórisdóttir Ó, Ólafsdottir E, et al. Individual risk assessment and information technology to optimise screening frequency for diabetic retinopathy. 2011; **54**(10): 2525.

4. Semeraro F, Parrinello G, Cancarini A, et al. Predicting the risk of diabetic retinopathy in type 2 diabetic patients. *J Diabetes Complications* 2011; **25**(5): 292-7.

5. Mehlsen J, Erlandsen M, Poulsen PL, Bek T. Individualized optimization of the screening interval for diabetic retinopathy: a new model. *Acta Ophthalmol* 2012; **90**(2): 109-14.

6. Hayes AJ, Leal J, Gray AM, Holman RR, Clarke PM. UKPDS outcomes model 2: a new version of a model to simulate lifetime health outcomes of patients with type 2 diabetes mellitus using data from the 30 year United Kingdom Prospective Diabetes Study: UKPDS 82. *Diabetologia* 2013; **56**(9): 1925-33.

7. Harris Nwanyanwu K, Talwar N, Gardner TW, Wrobel JS, Herman WH, Stein JD. Predicting development of proliferative diabetic retinopathy. *Diabetes Care* 2013; **36**(6): 1562-8.

8. Tanaka S, Tanaka S, Iimuro S, et al. Predicting macro- and microvascular complications in type 2 diabetes: the Japan Diabetes Complications Study/the Japanese Elderly Diabetes Intervention Trial risk engine. *Diabetes Care* 2013; **36**(5): 1193-9.

9. Stratton IM, Aldington SJ, Taylor DJ, Adler AI, Scanlon PH. A simple risk stratification for time to development of sight-threatening diabetic retinopathy. *Diabetes Care* 2013; **36**(3): 580-5.

10. Lagani V, Chiarugi F, Thomson S, et al. Development and validation of risk assessment models for diabetes-related complications based on the DCCT/EDIC data. *J Diabetes Complications* 2015; **29**(4): 479-87.

11. McEwan P, Bennett H, Ward T, Bergenheim K. Refitting of the UKPDS 68 risk equations to contemporary routine clinical practice data in the UK. *Pharmacoeconomics* 2015; **33**(2): 149-61.

12. Scanlon PH, Aldington SJ, Leal J, et al. Development of a cost-effectiveness model for optimisation of the screening interval in diabetic retinopathy screening. *Health Technol Assess* 2015; **19**(74): 1-116.

13. Hippisley-Cox J, Coupland C. Development and validation of risk prediction equations to estimate future risk of blindness and lower limb amputation in patients with diabetes: cohort study. *Bmj* 2015; **351**: h5441.

14. Basu S, Sussman JB, Berkowitz SA, Hayward RA, Yudkin JS. Development and validation of Risk Equations for Complications Of type 2 Diabetes (RECODe) using individual participant data from randomised trials. *Lancet Diabetes Endocrinol* 2017; **5**(10): 788-98.

15. Eleuteri A, Fisher AC, Broadbent DM, et al. Individualised variable-interval risk-based screening for sight-threatening diabetic retinopathy: the Liverpool Risk Calculation Engine. *Diabetologia* 2017; **60**(11): 2174-82.

16. Dagliati A, Marini S, Sacchi L, et al. Machine Learning Methods to Predict Diabetes Complications. *J Diabetes Sci Technol* 2018; **12**(2): 295-302.

17. García-Fiñana M, Hughes DM, Cheyne CP, et al. Personalized risk-based screening for diabetic retinopathy: A multivariate approach versus the use of stratification rules. *Diabetes Obes Metab* 2019; **21**(3): 560-8.

18. Ochs A, McGurnaghan S, Black MW, et al. Use of personalised risk-based screening schedules to optimise workload and sojourn time in screening programmes for diabetic retinopathy: A retrospective cohort study. *PLoS Med* 2019; **16**(10).

19. Fay MP SP. Exact and Asymptotic Weighted Logrank Tests for Interval Censored Data: The interval R Package. Journal of Statistical Software; 2010.

20. C A-B. icenReg: Regression Models for Interval Censored Data in R.: Journal of Statistical Software; 2017.
